# Integrated Differential Expression Analysis and WGCNA Identify Hub Genes Underlying Cotton Plant Height Development

**DOI:** 10.3390/ijms27114967

**Published:** 2026-05-30

**Authors:** Ruiqiang Qi, Juwu Gong, Yangming Liu, Haoliang Yan, Wankui Gong, Haihong Shang, Youlu Yuan, Quanjia Chen

**Affiliations:** 1 Engineering Research Centre of Cotton, Ministry of Education, College of Agriculture, Xinjiang Agricultural University, Urumqi 830052, Chinagongjuwu@caas.cn (J.G.); 2 State Key Laboratory of Cotton Bio-Breeding and Integrated Utilization, Institute of Cotton Research, Chinese Academy of Agricultural Sciences, Anyang 455000, China

**Keywords:** plant height, dynamic development, transcriptome, WGCNA, hub genes

## Abstract

Plant height is a key agronomic trait that influences plant architecture and mechanical harvesting suitability in cotton; however, the molecular mechanisms underlying its dynamic development remain unclear. In this study, two recombinant inbred line (RIL) populations sharing CCRI127 as a common paternal parent (RIL-GH07, *n* = 150; RIL-2358B, *n* = 276) were developed. Based on stable plant-height performance across multiple environments, tall and short extreme lines were selected from the two RIL populations for transcriptome sequencing. By integrating differential expression analysis with weighted gene co-expression network analysis (WGCNA), we identified hub genes associated with cotton plant height development, characterized the molecular features and core pathways governing dynamic stem elongation at different growth stages, thereby providing insights into the transcriptional regulation of plant height development in cotton. The two RIL populations showed broadly similar plant-height growth patterns, with slow elongation at 15 DOS, rapid elongation during 30–60 DOS, and reduced growth after 70 DOS. Transcriptome differential expression analysis identified 15,052 non-redundant DEGs, which exhibited clear population- and stage-specific expression patterns. In the GH07 population, the largest number of DEGs was detected at 15 DOS (7193), whereas in the 2358B population relatively large numbers of DEGs were maintained at both 30 DOS (3839) and 70 DOS (3118). Analysis of DEGs shared by the two populations across four developmental stages showed that, in addition to genes with consistent expression trends, each stage also contained a substantial number of DEGs with opposite expression directions. WGCNA identified 25 gene expression modules, among which the green and yellow modules were significantly positively correlated with plant height. Functional enrichment analysis indicated that genes in these two modules were mainly enriched in hormone regulation and signal transduction, protein modification and degradation, and intracellular transport. Seven hub genes were identified by integrating intramodular connectivity and kME values. Functional prediction suggested that these genes may play important roles in cotton plant height development. This study provides genetic resources and a theoretical basis for subsequent functional validation of cotton plant height-related genes and the improvement of plant architecture in cotton.

## 1. Introduction

Cotton (*Gossypium* spp.) is the world’s most important natural fibre crop and an important source of cottonseed oil [1]. One of the major goals of modern crop breeding is to optimize plant architecture to develop varieties that are suitable for intensive agricultural production and exhibit strong environmental adaptability [2]. Plant height is a key trait regulating cotton canopy architecture, which affects ventilation and light interception, lodging resistance and adaptation to mechanical harvesting [3,4,5]. Previous studies have shown that plant height is closely associated with fruiting branch arrangement, planting density and other factors, and directly affects cotton yield formation and field management efficiency [3].

Plant height has long been a central focus of genetic improvement in gramineous crops, and substantial theoretical knowledge on the mechanisms underlying plant height formation has accumulated in multiple species. The discovery and application of the Green Revolution gene *SD1* in rice and the *Rht* dwarfing genes in wheat laid a theoretical and practical foundation for modern crop architecture improvement and lodging-resistance breeding [6,7]. In recent years, transcriptomic studies of plant height have progressed from the previous pairwise comparisons involving only single material, single time point or a specific elongating tissue to systematic analyses based on continuous sampling across multiple stages and temporal expression networks. Chen et al. performed transcriptome sequencing and weighted gene co-expression network analysis (WGCNA) on a tall rice mutant and the wild type at the jointing and heading stages, and screened 11 candidate genes involved in auxin- and cytokinin-related regulation of plant height [8]. Time-course transcriptome analysis of deepwater rice under flooding revealed the dynamic regulatory pattern of rapid internode elongation and confirmed that genes such as *SK1/2* and *OsGA20ox2/SD1*, together with ethylene, gibberellin and jasmonate metabolic pathways and cell-wall remodeling processes, constitute major molecular bases driving internode elongation and plant height variation [9]. Wang et al. constructed hybrids from three maize lines with contrasting plant height and, through multi-stage transcriptome analysis, identified candidate genes related to internode cell regulation, cell-wall biosynthesis, and gibberellin and brassinosteroid signal transduction [10]. Cheng et al. carried out a high-temporal-resolution transcriptome analysis of maize stem development and found that the expression of NAC- and MYB-family transcription factors, together with genes associated with phenylpropanoid metabolism and flavonoid biosynthesis, changed continuously during development [11]. These studies indicate that plant height-related processes, including stem elongation, cell wall remodeling, and tissue formation, exhibit clear developmental stage specificity, and that their regulatory mechanisms are characterized by a dynamic transcriptional regulatory network involving the coordinated participation of multiple pathways.

At present, studies on the genetic regulation of cotton plant height have also made notable progress. Using backcross introgression lines derived from upland cotton and sea island cotton, Ma et al. mapped a stable plant-height QTL interval, qPH-Dt1-1, and identified the auxin efflux carrier gene *GhPIN3*; functional verification showed that this gene negatively regulates cotton plant height [12]. Based on an upland cotton RIL population, Wu et al. detected 60 plant height-related QTLs across nine environments, revealed pronounced dynamic genetic effects across developmental stages, and screened 14 candidate genes [13]. Wang et al. combined histological, phytohormone and transcriptome analyses of the second elongating internode of cotton seedlings following mepiquat chloride treatment and found that gibberellin is the core hormone regulating growth inhibition by mepiquat chloride, while brassinosteroid, auxin, ethylene metabolism and signal transduction also participate in this process in a gibberellin-dependent manner [14]. An et al. performed integrated mRNA and miRNA sequencing of shoot apices using the upland cotton wild type *Ari971*, the dwarf mutant *Ari1327*, and the tall mutant *Ari3697*, and found that *miR166*, *miR172*, *miR828*, *miR858*, *miR159*, and their target genes may be involved in plant height regulation [15]. Another study compared two upland cotton cultivars with different sensitivities to mepiquat chloride and conducted transcriptome analysis at 1, 3, and 6 days after treatment. The results indicated that differential expression of genes involved in gibberellin and brassinosteroid biosynthesis, plant hormone signal transduction, and related transcription factors may constitute an important molecular basis for the contrasting responses of different cultivars to mepiquat chloride [16]. Using WGCNA, Huang et al. screened 10 key genes regulating cotton plant height development and found that oxidoreductase activity, cutin, suberin and wax biosynthesis, and photosynthesis-related pathways may all be associated with plant height regulation [17]. Using an improved weighted gene co-expression network analysis, Liu et al. identified a key candidate gene cluster that may regulate cotton fiber development [18]. In addition, Liang et al. used a gibberellin-sensitive extreme dwarf mutant for transcriptome analysis and demonstrated that differentially expressed genes related to gibberellin biosynthesis and signal transduction participate in the formation of the dwarf phenotype [19]. These findings indicate that hormone crosstalk, cell elongation, cell-wall biosynthesis and protein homeostasis collectively participate in the regulation of cotton plant height development. However, existing studies have mainly focused on QTL mapping, exogenous regulation, single mutants, or single-time-point analyses. In contrast, the molecular regulatory networks underlying the dynamic development of cotton plant height throughout the growth period in genetically segregating populations remain poorly understood.

In this study, the high-quality cotton cultivar CCRI127 [20] was used as the paternal parent to construct two upland cotton recombinant inbred line populations with marked phenotypic divergence. Stable tall and short extreme lines were selected from each population across multiple environments and subjected to comparative time-course transcriptome analysis at different developmental stages. By combining differentially expressed gene (DEG) analysis with weighted gene co-expression network analysis (WGCNA), this study identified regulatory modules and hub genes potentially associated with plant height development, with the aim of elucidating the transcriptional regulatory mechanisms underlying dynamic plant height development in high-quality upland cotton. These findings provide a theoretical basis and candidate gene resources for breeding cotton with ideal plant architecture and for molecular design-based improvement.

## 2. Results

### 2.1. Plant Height Phenotypes

**Figure 1:**
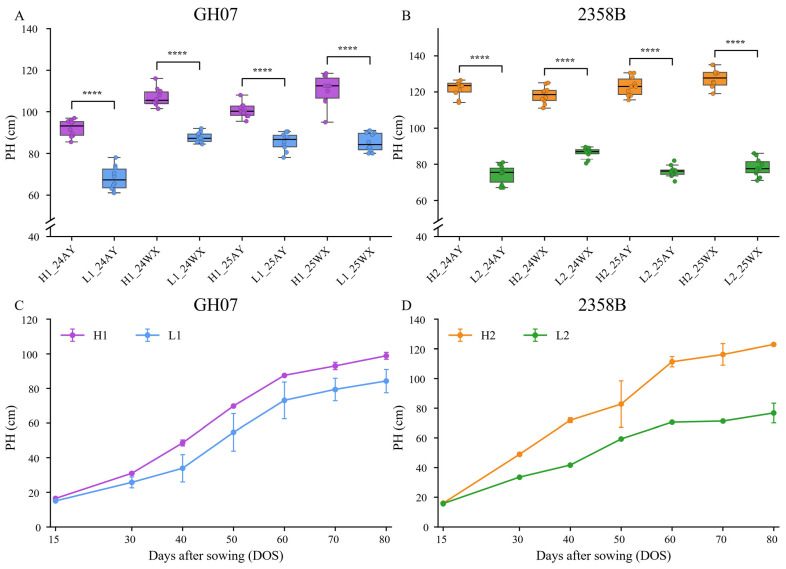
Differences in plant height among extreme lines across environments and their dynamic changes during developmental stages. (**A**) Comparison of plant height between the tall line H1 and the short line L1 from the GH07 population under four environments: Anyang 2024, Weixian 2024, Anyang 2025, and Weixian 2025. (**B**) Comparison of plant height between the tall line H2 and the short line L2 from the 2358B population under four environments: Anyang 2024, Weixian 2024, Anyang 2025, and Weixian 2025. (**C**) Dynamic changes in plant height of the extreme lines from the GH07 population across different growth and developmental stages. (**D**) Dynamic changes in plant height of the extreme lines from the 2358B population across different growth and developmental stages. Error bars represent the mean ± SE. **** indicates an extremely significant difference (*p* < 0.0001).

Tall line H1 and short line L1 from the GH07 population, together with tall line H2 and short line L2 from the 2358B population, showed significant and consistent differences in plant height across four environments: 24AY, 24WX, 25AY, and 25WX (Figure 1A,B). The plant-height differences between H1 and L1 were 23.16, 18.89, 14.50, and 26.11 cm in the four environments, respectively, whereas those between H2 and L2 were 48.94, 32.89, 47.11, and 48.28 cm, respectively. These results indicate that the phenotypes of the extreme lines were stable across years and locations. Analysis of dynamic changes in plant height across developmental stages showed that 15 DOS represented the initial phase of stem elongation, when all materials were relatively short and growth was slow, and differences between tall and short lines were not yet obvious. From 30 to 60 DOS, plant height entered a rapid elongation phase, and differences among materials progressively widened. By the late elongation stage (70 DOS), plant height growth slowed again and differences among materials became largely stable (Figure 1C,D). On the basis of the multi-environment phenotypic comparison and developmental dynamic analysis, 15, 30, 40 and 70 DOS were identified as key stages of cotton plant height development for transcriptome sampling and analysis.

### 2.2. Sequencing Data Analysis and Correlation Analysis of Biological Replicates

After low-quality sequences were removed, each library yielded an average of approximately 6.03 × 10^7^ clean reads (range 3.71 × 10^7^–7.94 × 10^7^) (Supplementary Table S1). Mapping statistics showed that the proportion of uniquely mapped reads (Unique Reads Percent) averaged approximately 92.57% (86.54–94.25%).

**Figure 2:**
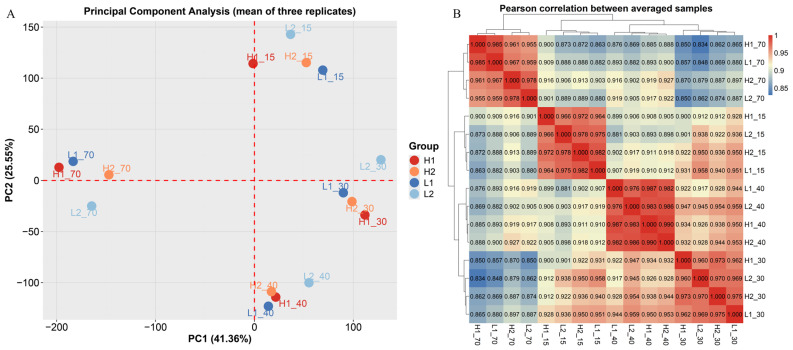
Principal component analysis and sample correlation analysis of RNA-seq samples. (**A**) Principal component analysis of the RNA-seq samples. (**B**) Pearson correlation clustering heat map among the RNA-seq samples.

The high unique mapping rate indicated that most clean reads were reliably aligned to the reference genome and that the RNA-seq data met the quality requirements for subsequent analyses. Based on the count matrix, genes with counts ≥10 in at least three samples were considered expressed in this study, resulting in 56,880 expressed genes across all samples. PCA showed a relatively clear separation of the 48 samples according to developmental stage (Figure 2A). The Pearson correlation clustering heat map showed that all replicate samples had correlation coefficients above 0.9 (Figure 2B), and therefore no samples were removed. These results indicate that the transcriptome sequencing data constructed in this study were reliable and suitable for downstream analyses.

### 2.3. Stage- and Population-Specific Differential Expression Patterns in Extreme Plant-Height Materials

**Figure 3:**
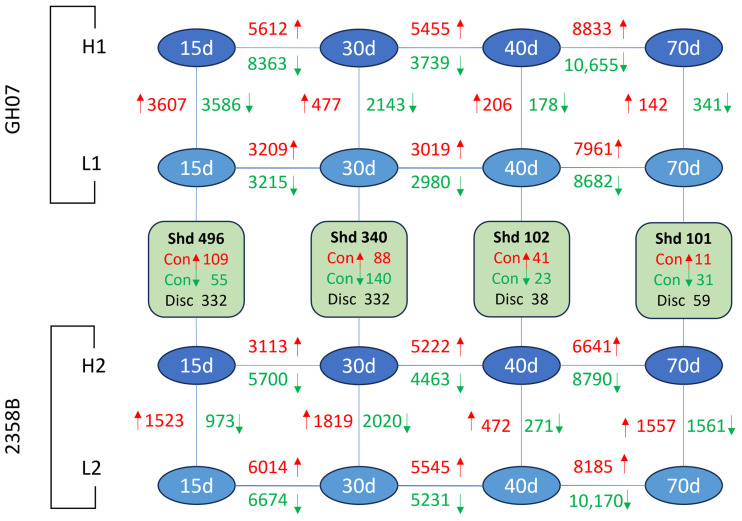
Differential expression analysis of extreme materials from the two populations. Note: H and L denote the tall and short lines, respectively. Red numbers and upward arrows indicate the numbers of up-regulated DEGs, whereas green numbers and downward arrows indicate the numbers of down-regulated DEGs. Shd denotes the number of DEGs shared by the two populations at the same stage; Con denotes the number of shared DEGs with consistent expression direction; Disc denotes the number of shared DEGs with inconsistent expression direction.

As shown in Figure 3, the extreme plant-height materials from the two RIL populations exhibited marked differences in gene expression patterns at the same developmental stage. In the GH07 population, 3607, 477, 206 and 142 up-regulated genes, and 3586, 2143, 178 and 341 down-regulated genes, were detected between H1 and L1 at 15, 30, 40 and 70 DOS, respectively. In the 2358B population, 1523, 1819, 472 and 1557 up-regulated genes, and 973, 2020, 271 and 1561 down-regulated genes, were detected between H2 and L2 at 15, 30, 40 and 70 DOS, respectively. Overall, the magnitude and direction of differential expression were not consistent between the two populations across developmental stages, suggesting that transcriptomic regulation associated with plant height may show pronounced stage specificity under different genetic backgrounds. Further joint analysis of DEGs from the two populations at the same stage identified 496, 340, 102 and 101 shared DEGs at 15, 30, 40 and 70 DOS, respectively. Analysis of these shared DEGs across the four developmental stages showed that, in addition to genes with concordant expression trends, each stage also contained a set of significant DEGs with opposite expression directions.

### 2.4. Functional Enrichment Analysis of Differentially Expressed Genes

GO and KEGG enrichment analyses were performed for the differentially expressed genes (DEGs) at different developmental stages. To facilitate the presentation of the results, only the top 10 most significant GO and KEGG terms in each group were selected for visualization (Supplementary Figure S1). Overall, the GO and KEGG enrichment results showed a high degree of consistency. The DEGs were mainly associated with photosynthesis, chloroplast organization and chloroplast function-related processes, pigment metabolism, phenylpropanoid metabolism and biosynthesis, flavonoid biosynthesis, defense response, glutathione metabolism, and the plant MAPK signaling pathway. These results indicate that the formation of plant height differences is not determined by a single process, but is jointly regulated by multiple biological processes, including photosynthesis, secondary metabolism, defense response, antioxidant regulation, and signal transduction.

In addition, the functional enrichment patterns differed markedly between the two populations. In the GH07 population, the enrichment results at different developmental stages showed a relatively clear stage-specific pattern. At the early elongation stage, the DEGs were mainly enriched in processes such as photosynthesis and chloroplast organization. During the rapid elongation stage, the enriched terms gradually shifted toward signal response-related processes, including the jasmonic acid-mediated signaling pathway, response to salicylic acid, and the plant MAPK signaling pathway. At the late elongation stage, the DEGs were mainly enriched in terms such as plant-type secondary cell wall biogenesis, lignin biosynthetic process, and phenylpropanoid biosynthetic process. In contrast, in the 2358B population, the DEGs at the early elongation stage were mainly enriched in secondary metabolism-related processes, including terpene metabolic process, flavonoid biosynthetic process, and phenylpropanoid biosynthesis. During the rapid elongation stage, the DEGs were enriched in photosynthesis, light harvesting, pigment biosynthetic process, and the plant MAPK signaling pathway. At the late elongation stage, they were mainly enriched in pathways such as secondary metabolite biosynthetic process, phenylpropanoid biosynthesis, brassinosteroid biosynthesis, and starch and sucrose metabolism.

These analyses further indicate that the enrichment patterns of DEGs in the two RIL populations showed both common features and clear population specificity. The GH07 population showed a stronger association with photosynthesis and structural formation, whereas the 2358B population was more closely related to secondary metabolism, defense response, and signal regulation. These findings suggest that plant height formation under different genetic backgrounds may be regulated by different functional modules.

### 2.5. Gene Co-Expression Network Analysis

**Figure 4:**
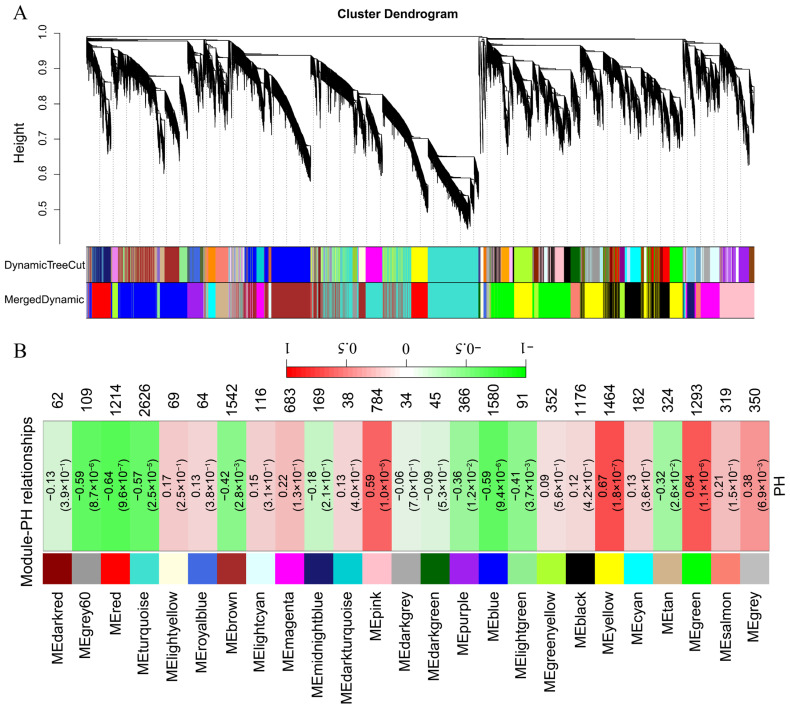
Weighted gene co-expression network analysis (WGCNA) of differentially expressed genes. (**A**) Gene clustering dendrogram and module assignment based on the topological overlap matrix (TOM). The upper panel shows the gene clustering tree, whereas the lower panels show the module colours obtained by dynamic tree cutting and after module merging, respectively. (**B**) Heat map of correlations between module eigengenes and plant height (PH). The values shown in the cells are correlation coefficients, with significance *p*-values given in parentheses; red indicates positive correlation, green indicates negative correlation, and the numbers above the heat map indicate the number of genes in each module.

To investigate the relationship between gene expression and plant height development and to screen core genes associated with plant height development, we constructed a WGCNA co-expression network using 15,052 non-redundant DEGs. According to the scale-free topology criterion, a soft-thresholding power of 11 was selected as the optimal value, and a scale-free network with average connectivity approaching 0 was subsequently constructed (Supplementary Figure S2). A total of 25 co-expression modules were obtained (Figure 4A), among which the turquoise module contained the largest number of genes (2626) and the dark turquoise module the fewest (34); genes that could not be assigned to any module were placed in the grey module (350; Figure 4B). Among the 25 modules obtained, 12 were negatively correlated and 13 were positively correlated with plant height. Using |r| ≥ 0.6 and *p*-value < 0.01 as thresholds, two modules showing significant positive correlations with cotton plant height were identified: the yellow module (r = 0.67, *p* = 1.1 × 10^−6^) and the green module (r = 0.64, *p* = 1.8 × 10^−7^). Genes in these two modules may therefore be associated with cotton plant height development.

#### 2.5.1. Functional Enrichment Analysis of Cotton Plant Height-Specific Modules

**Figure 5:**
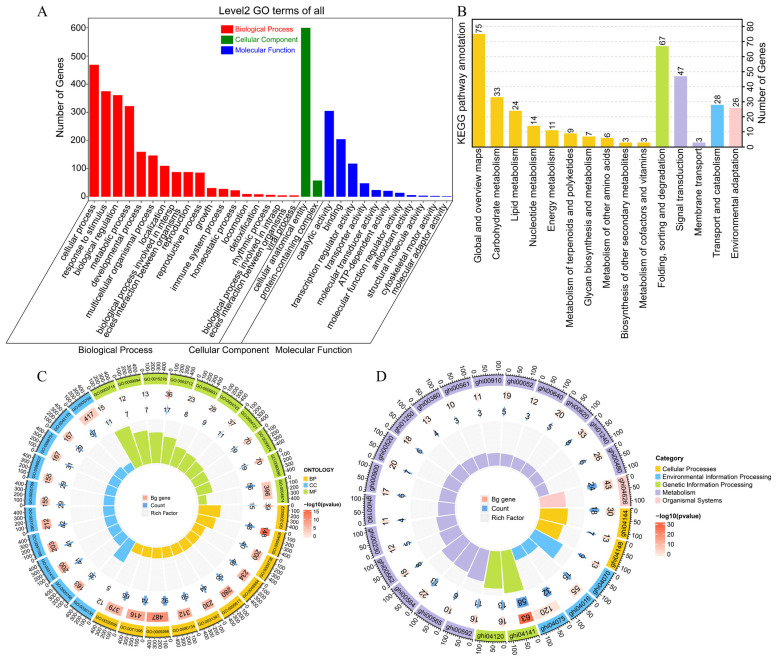
GO and KEGG enrichment analyses of genes in the green module. (**A**) Level 2 GO classification of genes in the green module. (**B**) KEGG pathway classification of genes in the green module. (**C**) Top 10 enriched GO terms in each of the Biological Process (BP), Cellular Component (CC), and Molecular Function (MF) categories for genes in the green module. (**D**) Top 25 significantly enriched KEGG pathways for genes in the green module.

**Figure 6:**
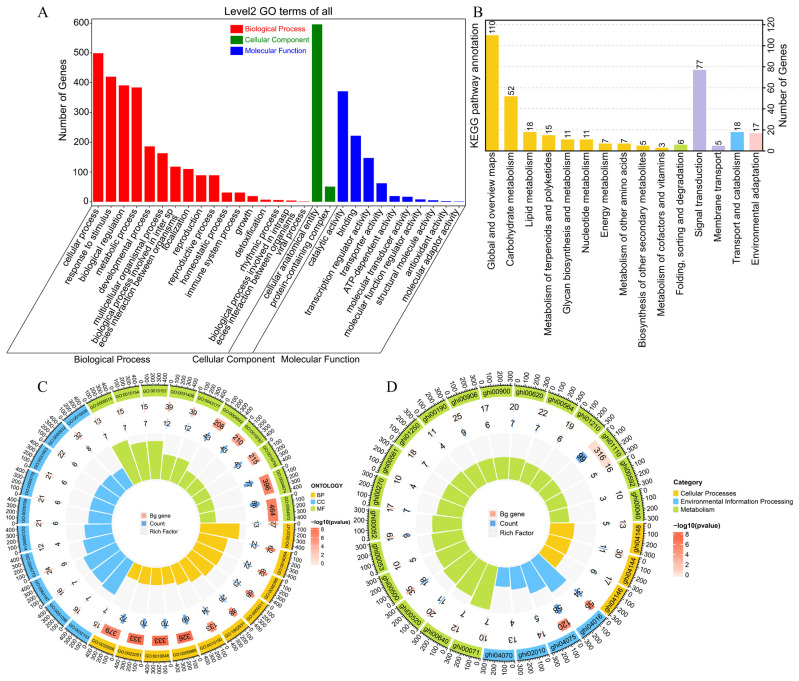
GO and KEGG enrichment analyses of genes in the yellow module. (**A**) Level 2 GO classification of genes in the yellow module. (**B**) KEGG pathway classification of genes in the yellow module. (**C**) Top 10 enriched GO terms in each of the Biological Process (BP), Cellular Component (CC), and Molecular Function (MF) categories for genes in the yellow module. (**D**) Top 25 significantly enriched KEGG pathways for genes in the yellow module.

To further elucidate the functions of genes within the target modules, GO and KEGG enrichment analyses were performed for the two modules that were significantly positively correlated with plant height, and the results are shown in Figure 5 and Figure 6.

GO enrichment analysis showed that the differentially expressed genes (DEGs) in both modules were mainly enriched in the three major GO categories, namely biological process (BP), molecular function (MF), and cellular component (CC) (Figure 5A and Figure 6A). The GO enrichment analysis of the green module showed that genes in this module were mainly enriched in the BP category for the regulation of jasmonic acid-mediated signaling pathway, abscisic acid-activated signaling pathway, and intracellular signal transduction, as well as processes related to stress responses and hormone signaling regulation (Figure 5C). In the CC category, these genes were mainly enriched in membrane-bound organelle-related structures, including the Golgi apparatus, vesicle, endosome, and endoplasmic reticulum membrane. In the MF category, they were mainly enriched in protein serine/threonine kinase activity, phosphoprotein phosphatase activity, and calcium-dependent protein serine/threonine kinase activity.

The enrichment analysis of the yellow module showed that genes in this module were mainly enriched in the BP category for intracellular signal transduction, regulation of signal transduction, and activation of protein kinase activity (Figure 6C). In the CC category, these genes were mainly enriched in the late endosome, cell division site, cell tip, and cell pole. In the MF category, they were significantly enriched in protein kinase activity, protein serine/threonine kinase activity, and ubiquitin-protein transferase activity.

The KEGG classification analysis showed that genes in the green module were mainly distributed in categories related to folding, sorting and degradation, and signal transduction (Figure 5D), whereas genes in the yellow module were mainly distributed in categories related to signal transduction and carbohydrate metabolism (Figure 6B). Further analysis of significantly enriched pathways showed that genes in the green module were significantly enriched in protein processing in endoplasmic reticulum, ubiquitin-mediated proteolysis, and alpha-linolenic acid metabolism (Figure 5D). In contrast, genes in the yellow module were significantly enriched in the plant MAPK signaling pathway and plant hormone signal transduction (Figure 6D).

#### 2.5.2. Construction of Gene Interaction Networks and Identification of Hub Genes

**Figure 7:**
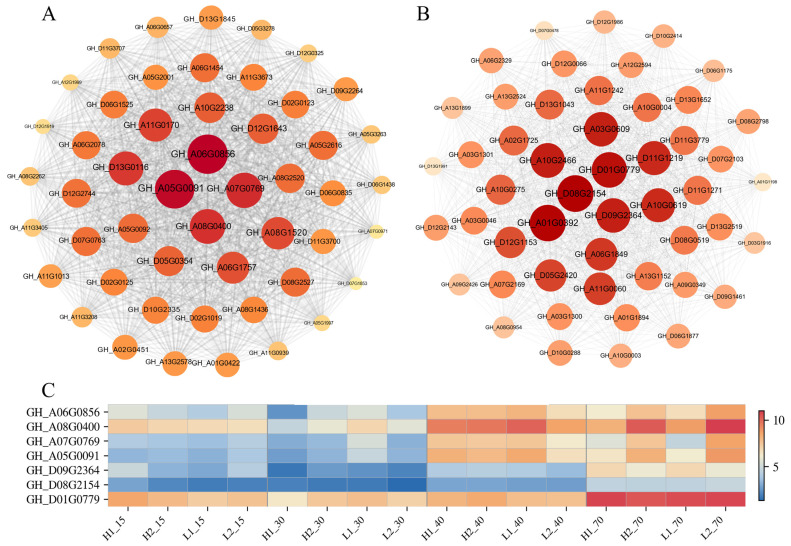
Co-expression networks of hub genes from plant height-related modules and analysis of their expression patterns. (**A**) Co-expression network of hub genes in the green module. (**B**) Co-expression network of hub genes in the yellow module. (**C**) Heatmap showing the expression patterns of seven hub genes across different materials and developmental stages. The x-axis represents different samples and sampling stages, and the y-axis represents the hub genes. Heatmap colours indicate gene expression levels, with blue representing low expression and red representing high expression. Expression levels were calculated as the mean values of three biological replicates and visualised after log2(value + 1) transformation.

To identify hub genes potentially involved in plant height regulation within these two modules, the top 50 genes ranked by intramodular connectivity were used to construct co-expression networks, from which seven hub genes were identified (Figure 7A,B). Four hub genes, *GH_A06G0856*, *GH_A08G0400*, *GH_A07G0769* and *GH_A05G0091*, were identified in the green module; among them, *GH_A06G0856* and *GH_A07G0769* were shared DEGs between the two populations, whereas *GH_A08G0400* and *GH_A05G0091* were differentially expressed only in the GH07 population. Functional annotation indicated that these genes are involved in jasmonic acid signaling and biosynthesis, lipid signal transduction, RNA silencing and the transition of vegetative developmental phase, respectively. Three hub genes, *GH_D09G2364*, *GH_D08G2154* and *GH_D01G0779*, were identified in the yellow module, and all three were differentially expressed only in the GH07 population. Functional annotation indicated that these genes are mainly involved in protein kinase-mediated signal transduction, cytoskeleton-related regulation and protein translation initiation. Further analysis of the expression patterns of these seven core genes in the four extreme materials (Figure 7C) showed that they exhibited highly consistent dynamic changes. Their expression levels were low at 15 DOS, began to increase gradually at 30 DOS, and reached the highest levels at 70 DOS.

### 2.6. qRT-PCR Validation of Candidate Hub Gene Expression Patterns

To validate the expression patterns of the hub genes and the reliability of the RNA-seq results, qRT-PCR was performed to examine the expression of seven hub genes in this study (Supplementary Figure S3). The results showed that the qRT-PCR expression trends of these genes across different materials and developmental stages were generally consistent with the expression changes observed in the transcriptome sequencing analysis, indicating the high reliability of the RNA-seq data in this study.

## 3. Discussion

Cotton plant height is an important trait affecting mechanical harvesting, lodging resistance and yield formation, and its development is jointly regulated by multiple processes, including internode number, internode elongation and apical meristem growth [21,22,23]. In this study, extreme lines were selected from two RIL populations sharing a common paternal parent and were used for further analysis. Phenotypic evaluation showed highly significant differences in plant height among the extreme lines across two years and two locations. The dynamic phenotypic changes observed at 15, 30, 40, 50, 60, 70, and 80 days after sowing in Anyang in 2025 showed clear stage-specific differentiation. Previous time-series transcriptomic and developmental biology studies have shown that changes in gene expression often precede visible morphological changes or the accumulation of tissue components [24,25]. Therefore, 15, 30, 40, and 70 days after sowing may represent key stages influencing shoot apex differentiation in cotton. Differential expression analysis showed that the GH07 population had the largest number of differentially expressed genes at 15 d, with 7193 DEGs, followed by a marked decrease after 30 d. In the 2358B population, relatively large numbers of DEGs were retained at both 30 d and 70 d, whereas the fewest DEGs were detected at 40 d. Analysis of the DEGs shared by the two populations at the same developmental stage showed that 496, 340, 102, and 101 common DEGs were identified at 15, 30, 40, and 70 d, respectively. At each stage, genes with consistent and opposite expression directions coexisted. Functional enrichment analysis of the DEGs revealed both shared and population-specific functional features between the two populations. Overall, the shared DEGs were mainly involved in processes such as photosynthesis, pigment metabolism, and phenylpropanoid metabolism and biosynthesis. In terms of population specificity, DEGs in the GH07 population were more prominently associated with photosynthesis during the early elongation stage and with structural formation during the late elongation stage, whereas those in the 2358B population were more closely related to secondary metabolism, defense responses, and signal regulation. These results were consistent with the differential expression analysis, suggesting that plant height formation under different genetic backgrounds may involve distinct functional pathways or transcriptional modules. Previous studies have identified many stable QTLs through linkage analysis and association analysis [12,13,26,27]. However, comparative analysis showed that the hub genes identified in this study were not located within the previously reported QTL intervals. This may be attributable to the use of experimental materials different from those used in previous studies, as well as the application of WGCNA to identify candidate genes.

In this study, WGCNA was performed based on the non-redundant DEGs identified at different developmental stages in the two populations, and the results showed that the green and yellow modules were significantly positively correlated with cotton plant height. Genes in these modules were mainly distributed in regulatory networks associated with hormone signaling, membrane lipid signaling and transcriptional regulation. In the green module, the identified genes encoding a JAZ/TIFY protein (*GH_A06G0856*) and *AOC4* (*GH_A08G0400*) are both important components of the jasmonic acid (JA) pathway [28,29]. Previous studies have shown that the JA pathway plays a key role in coordinating plant growth inhibition and defence responses, and that its effects on plant growth and development are largely mediated through crosstalk with gibberellin (GA) and other hormone signaling pathways. The antagonistic relationship between JAZ and DELLA proteins is considered an important molecular basis of JA-GA crosstalk [30]. In rice, *OsJAZ9* can directly interact with the DELLA protein SLR1; overexpression of *OsJAZ9* enhances GA responses, whereas knockout weakens them [31]. *DGK1*-like proteins (*GH_A07G0769*) are membrane lipid signaling enzymes involved in plant development and stress responses. In *Arabidopsis*, *DGK2* and *DGK4* participate not only in gametophyte development but also in vegetative growth, and suppression of their function leads to rounder leaves, smaller seedlings and reduced root length [32]. *SGS3* (*GH_A05G0091*) is a key component of ta-siRNA biogenesis and is involved in RNA silencing, the transition between vegetative developmental phases and temporal developmental regulation [33,34]. *TAS3*-derived ta-siRNAs can regulate auxin response factors such as *ARF3* and *ARF4*, thereby affecting organ formation and developmental patterning [35]. Notably, in poplar the conserved miR390-TAS3-ARF pathway has been shown to be directly associated with stem elongation and increases in plant height [36].

In the yellow module, the CBL-CIPK family member *GH_D09G2364* has been implicated in polar auxin transport and root growth. In *Arabidopsis*, *CIPK6* participates in polar auxin transport and root development, whereas *CIPK25* is associated with root meristem development [37]. In rice, *OsCBL8-OsCIPK17* regulates seed germination and seedling growth [38], indicating a close association between CIPK proteins and plant growth. NET proteins are a plant-specific family of actin-binding proteins that mainly establish connections between the cytoskeleton and membrane systems and play important roles in organelle positioning, membrane remodeling and intracellular structural coordination [39,40]. Because the actin cytoskeleton is closely linked to cell expansion, polar growth and tissue organisation, *GH_D08G2154* may be involved in cotton plant height development through its potential association with cytoskeleton organization, cell-shape maintenance and tissue elongation processes [41]. In addition, *GH_D01G0779* is annotated as being associated with the regulation of translation initiation in plants. This gene may provide a potential link between protein synthesis and growth-related processes during cotton plant height development [42].

## 4. Materials and Methods

### 4.1. Plant Materials

Two RIL populations were constructed using GH07-44 and 2358B-4 as the respective maternal parents and CCRI127 as the common paternal parent. These populations are hereafter referred to as the GH07 population (GH07-44 × CCRI127) and the 2358B population (2358B-4 × CCRI127), respectively [43,44]. Based on stable plant-height performance across multiple environments, one extremely tall line and one extremely short line were selected from each population: H1 and L1 from the GH07 population, and H2 and L2 from the 2358B population. These four extreme lines were used for subsequent transcriptome sequencing.

### 4.2. Field Planting

In 2024 and 2025, the four extreme lines were planted in Anyang, Henan (latitude 36°06′ N, longitude 114°20′ E) and Weixian, Hebei (latitude 36°59′04″ N, longitude 115°15′55″ E), representing four environments: 24AY, 25AY, 24WX and 25WX. The row length was 3.0 m, with a row spacing of 0.8 m and a plant spacing of 0.20–0.25 m. Field management was carried out according to local agricultural practices.

### 4.3. Phenotypic Evaluation

Plant height was evaluated as the vertical height from the cotyledonary node to the main stem growth point and was recorded in centimeters (cm); after topping, the vertical distance from the cotyledonary node to the apex of the main stem was defined as the final plant height [45]. Dynamic measurements of plant height were conducted at 15, 30, 40, 50, 60, 70 and 80 days after sowing (DOS). At each time point, 10 uniformly growing plants were selected from each line for measurement. The plant height recorded after topping was used as the final plant height to compare differences among the extreme lines.

### 4.4. RNA Extraction and Library Construction

At 15, 30, 40 and 70 DOS, young stem-apex tissues from the main stem were collected from four materials for transcriptome sequencing. For each material at each stage, three biological replicates were prepared, and each biological replicate consisted of pooled stem-apex tissues from 3–5 plants with uniform growth status. During sampling, approximately 0.5 cm of young apical tissue from the top of the main stem was excised from each plant, all samples were collected before cotton topping. In total, 48 samples were obtained (4 stages × 4 materials × 3 biological replicates). All samples were immediately frozen in liquid nitrogen after collection and stored at −80 °C until use.

Total RNA was extracted from the stem apex samples using the RNAprep Pure Plant Kit (TIANGEN Biotech, Beijing, China), and RNA integrity was assessed by 1% agarose gel electrophoresis. RNA concentration and purity were then determined using a NanoDrop 2000 spectrophotometer (Thermo Scientific, Waltham, MA, USA). Approximately 2 μg of total RNA from each sample was used for cDNA library construction. A total of 48 libraries were constructed and sequenced on the Illumina NovaSeq 6000 platform to produce 150-bp paired-end (PE) reads. The raw sequencing data were stored in FASTQ format and subjected to quality assessment using FastQC. Clean reads were obtained after removal of adaptor-contaminated and low-quality reads, and the GC content and Q30 values of the clean data were further calculated to evaluate whether the sequencing data met the requirements for downstream analyses.

### 4.5. Transcriptome Analysis

Reference genome indices were built using HISAT2 v2.0.5 [46], and clean reads were aligned to the *Gossypium hirsutum* TM-1_V2.1 reference genome [47]. Only uniquely mapped reads were retained and stored in BAM format. Gene expression was quantified using the featureCounts program in the Subread package [48], yielding a raw gene-count matrix for each sample together with corresponding gene-length information. Gene expression levels were represented using fragments per kilobase of transcript per million mapped reads (FPKM) values [49]. Pearson correlation analysis was used to assess consistency among samples. If the Pearson correlation coefficient among the three biological replicates was below 0.9, the corresponding sample was excluded from subsequent analyses.

### 4.6. Differential Expression Analysis

To systematically characterize the transcriptional differences between tall and short materials at different developmental stages, raw counts were used as the input for DESeq2 differential expression analysis in this study. The model design = ~ group was applied, with the extreme tall materials defined as the treatment group and the extreme short materials defined as the control group. Within each population, between-group comparisons were performed at the same developmental stage, and within-group comparisons were conducted between adjacent developmental stages. Principal component analysis (PCA) and Pearson correlation analysis were further performed based on the variance-stabilized expression matrix [50]. DEGs were screened using the following criteria: FDR < 0.05, |log2FoldChange| > 1, and counts ≥ 10 in at least three samples. Gene Ontology (GO) and Kyoto Encyclopedia of Genes and Genomes (KEGG) enrichment analyses of DEGs were then performed using the ClusterProfiler in R package, GO and KEGG terms with a *p*-value < 0.05 were considered significantly enriched. DEG annotations were based on homologous annotation from *Arabidopsis*.

### 4.7. Construction of Weighted Gene Co-Expression Networks and Screening of Hub Genes

To minimize noise from lowly expressed genes, subsequent analyses were performed using non-redundant DEGs identified at the same developmental stage [51]. The WGCNA in R package was used to perform WGCNA of DEGs. After variance-stabilizing transformation, the expression matrix was used as the input for network analysis. The minimum power corresponding to the first scale-free topology fit index (signed R^2^) reaching 0.80 was selected as the optimal soft-threshold power β. A signed network was used to construct the adjacency matrix, followed by calculation of the topological overlap matrix (TOM). Co-expression modules were identified on the basis of hierarchical clustering and dynamic tree cutting, with parameters set as minModuleSize = 30, deepSplit = 2 and mergeCutHeight = 0.25. Candidate hub genes within each module were comprehensively screened according to module membership (kME) and intramodular connectivity. In key modules, the top 50 genes ranked by intramodular connectivity were selected for network visualization in Cytoscape v3.10.4.

### 4.8. qRT-PCR Validation of Hub Genes

Reverse transcription was performed using the StarScript III All-in-one RT Mix with gDNA Remover kit (Kangrun Jingxing Suzhou Biotechnology Co., Ltd., Suzhou, China). The qRT-PCR reactions were conducted using ChamQ Universal SYBR qPCR Master Mix (Vazyme Biotech Co., Ltd., Nanjing, China). Quantitative real-time PCR primers were designed based on the CDS sequences of the candidate genes using the online Primer-BLAST tool (National Center for Biotechnology Information, Bethesda, MD, USA; Primer3 version 2.5.0). The primer sequences were synthesized by Shangya Biotechnology Co., Ltd. (Zheng, China) and are listed in Supplementary Table S2. The total reaction volume was 20 μL, and *GhActin7* was used as the reference gene. Three independent biological replicates were performed for each gene. The relative expression levels of the candidate genes were calculated using the 2^−ΔΔCt^ method [52].

## 5. Conclusions

In this study, transcriptome analysis was performed using cotton materials with stable plant-height differences at selected developmental stages. A total of 15,052 non-redundant DEGs were identified, and their expression patterns showed clear population- and stage-specific characteristics. Using weighted gene co-expression network analysis, the green and yellow modules were identified as being significantly associated with plant height. Functional enrichment analysis showed that genes in the green module were mainly associated with jasmonic acid/abscisic acid signaling, protein processing in the endoplasmic reticulum, ubiquitin-mediated proteolysis, and lipid metabolism, whereas the yellow module was mainly associated with the MAPK signaling pathway, plant hormone signal transduction, protein kinase activity, and cell polarity. Furthermore, seven candidate genes potentially involved in the regulation of plant height development were identified. However, because the transcriptome samples in this study were collected only from main-stem shoot apex tissues, the biological functions of the hub genes identified in cotton and their roles in cotton plant height regulation remain to be further investigated and validated. These results provide new evidence for further elucidating the molecular mechanisms underlying cotton plant height formation and provide a theoretical basis and gene resources for subsequent functional validation and plant architecture improvement.

## Data Availability

The original contributions presented in the study are included in the article/Supplementary Materials. Further inquiries can be directed to the authors.

## Supplementary Materials

The following supporting information can be downloaded at: https://www.mdpi.com/article/10.3390/ijms27114967/s1.

## Abbreviations

The following abbreviations are used in this manuscript:

RIL	Recombinant inbred line
DOS	Days after sowing
RNA-seq	RNA sequencing
DEG	Differentially expressed gene
WGCNA	Weighted gene co-expression network analysis
GO	Gene Ontology
KEGG	Kyoto Encyclopedia of Genes and Genomes
PCA	Principal component analysis
FPKM	Fragments per kilobase of transcript per million mapped reads
TOM	Topological overlap matrix
