# Supplementary material for: Integrated Differential Expression Analysis and WGCNA Identify Hub Genes Underlying Cotton Plant Height Development
Source: Int J Mol Sci. 2026 May 30;27(11):4967. doi: 10.3390/ijms27114967 (PMC13257404; doi:10.3390/ijms27114967)
Supplement: Supplementary file 1 [file Supplementary Figures.pdf › Supplementary Figures.pdf]

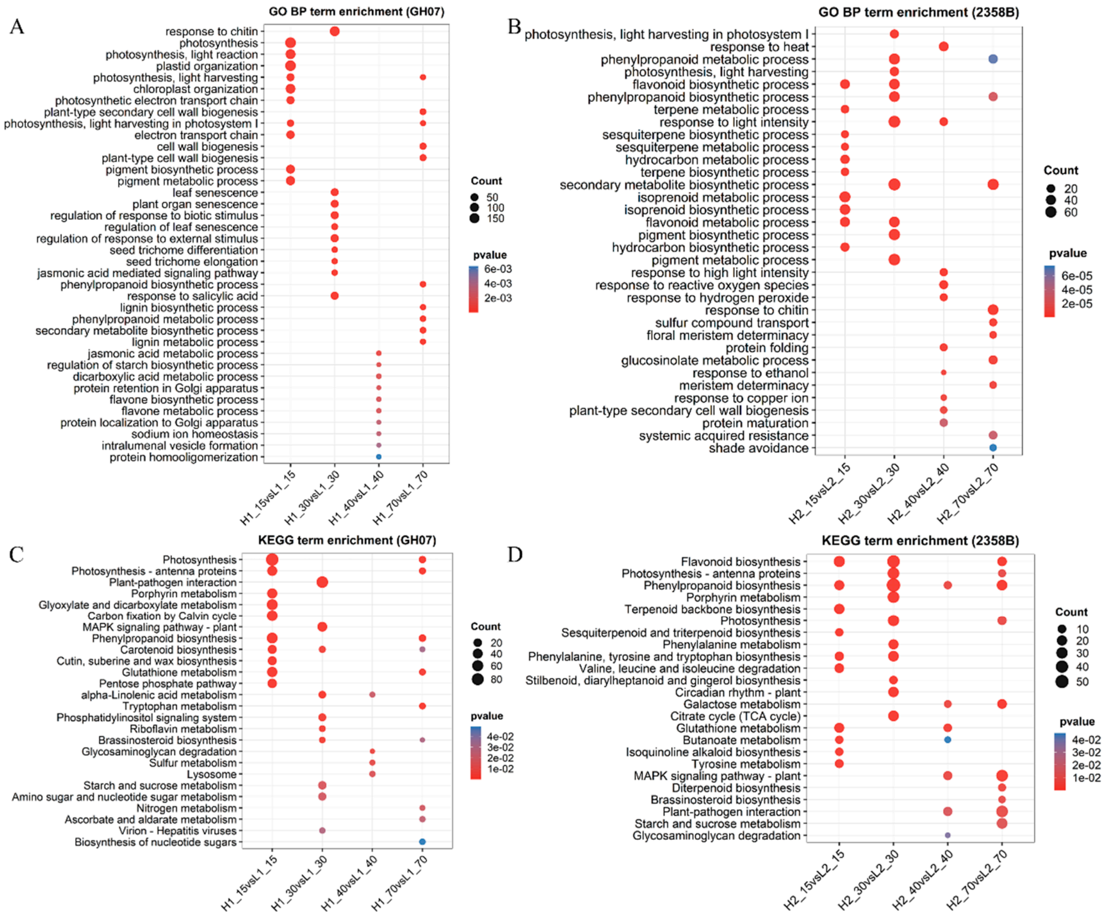

Supplementary Figure S1. GO and KEGG enrichment analyses of DEGs at different developmental stages in the two RIL populations. (A) GO biological process enrichment analysis of DEGs in the GH07 population. (B) GO biological process enrichment analysis of DEGs in the 2358B population. (C) KEGG pathway enrichment analysis of DEGs in the GH07 population. (D) KEGG pathway enrichment analysis of DEGs in the 2358B population.

Only the top 10 most significant terms or pathways are shown in each comparison. The x-axis represents comparisons between tall and short extreme lines at different developmental stages, and the y-axis represents enriched GO terms or KEGG pathways. Dot size indicates the number of DEGs enriched in each term or pathway, and dot colour represents the p-value.

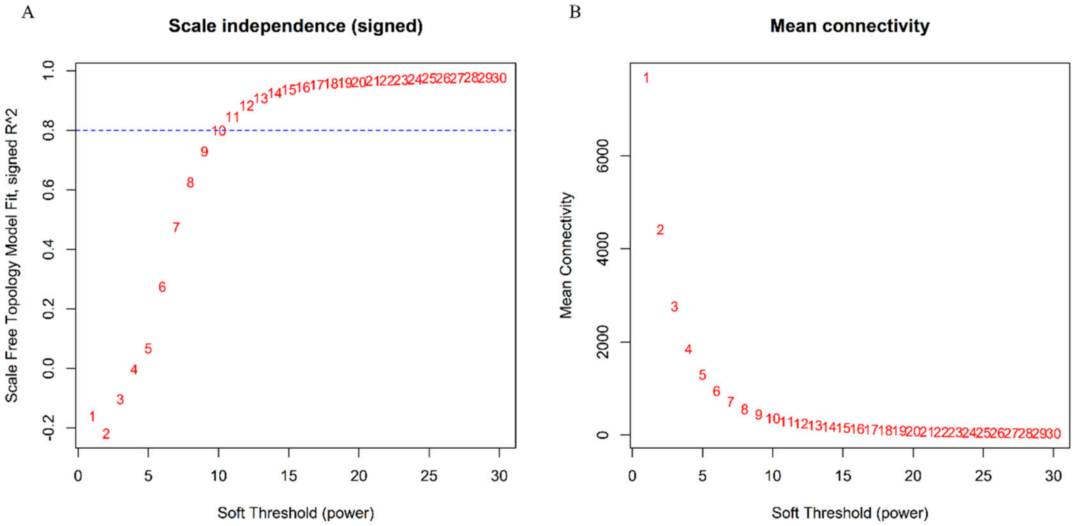

Supplementary Figure S2. Selection of the soft-thresholding power for WGCNA. (A) Scale-free topology fit index under different soft-thresholding powers. The blue dashed line indicates the threshold of signed $R^2 = 0.80$. (B) Mean connectivity under different soft-thresholding powers.

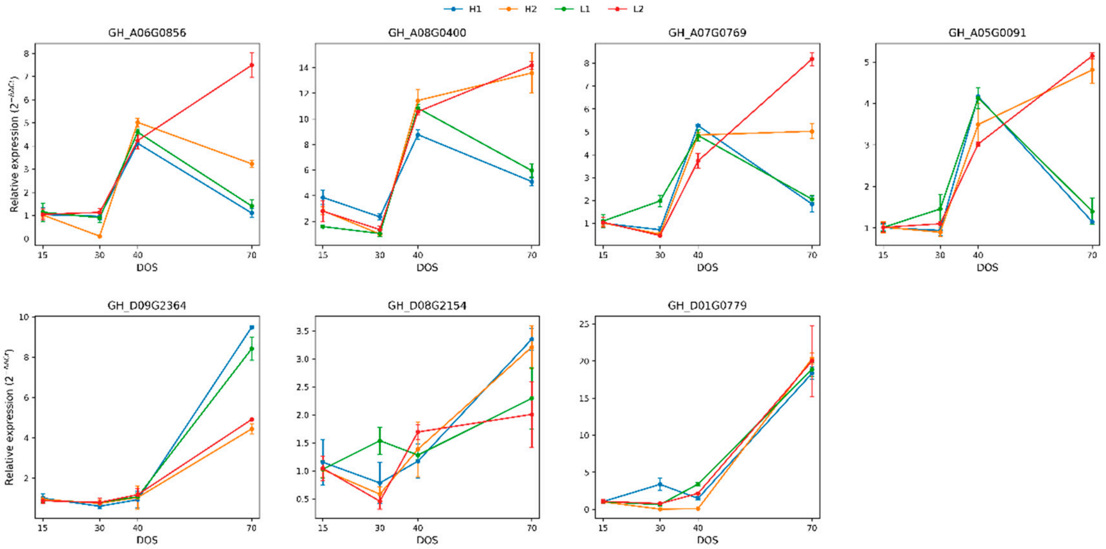

Supplementary Figure S3. qRT-PCR validation of seven candidate hub genes.

Expression patterns of seven candidate hub genes validated by qRT-PCR at different developmental stages. The seven genes include *GH_A06G0856*, *GH_A08G0400*, *GH_A07G0769*, *GH_A05G0091*, *GH_D09G2364*, *GH_D08G2154*, and *GH_D01G0779*. The x-axis represents developmental stages at 15, 30, 40, and 70 days after sowing (DOS), and the y-axis represents relative expression levels calculated using the $2^{-\Delta\Delta Ct}$ method. H1 and H2 represent tall lines from the GH07 and 2358B populations, respectively, whereas L1 and L2 represent short lines from the GH07 and 2358B populations, respectively. Error bars represent variation among biological replicates.
